# Water homeostasis gene expression in intestines of male and female broilers divergently selected and individually phenotyped for low and high water conversion ratio

**DOI:** 10.3389/fphys.2026.1954752

**Published:** 2026-10-16

**Authors:** Kentu Lassiter, Elizabeth S. Greene, Marciela Maqaeda, Travis Tabler, Kirsten Shafer, Brooklee Roach, Robert F. Wideman, Sami Dridi, Sara Orlowski-Workman, Walter G. Bottje

**Affiliations:** Center of Excellence for Poultry Science, Division of Agriculture, University of Arkansas, Fayetteville, AR, United States

**Keywords:** broilers, female, male, gene expression, genetic selection, intestines, water conversion ratio

## Abstract

**Background:**

The goal of this study was to evaluate gene expression of water homeostasis-related genes in the intestines of male and female broilers individually phenotyped for low and high water conversion ratio (LWCR and HWCR, respectively).

**Methods:**

A 2x2 factorial experiment was conducted with male and female broilers from fourth generation LWCR and HWCR lines. From 4 to 6 wk, broilers were housed in cages to phenotype birds for WCR (g water intake/g body weight gain [BWG]) and feed conversion ratio (FCR, g feed intake/BWG). At 6 wk, samples obtained from the upper duodenum, ileum, and cecum (n=5/group) were frozen in liquid nitrogen. Gene expression was determined by RT-PCR for: 1) the arginine vasotocin (*AVT*) system (*AVT*, mesotocin [*MT*] and receptors [*VR2, VR1a, VR1b*, *MTR*]), 2) the renin-angiotensin system (angiotensinogen [*AGT*], angiotensin receptors [*AT1, AT2*], renin [*REN*], *AGT* converting enzyme [*ACE*], and Na+/K+ ATPase [*ATP1B1*], 3) aquaporins (*AQP1–5* and 11), and 4) genes encoding tight junction proteins (occludin, [*OCLN*], and claudins [*CLDN2, CLDN15*]).

**Results:**

Gene (mRNA) expression of *AVT, VR1a, ACE*, and *AQP* 1,3 and 11 was lower and *CLDN2* higher in the LWCR compared to the HWCR line in the upper duodenum. In the ileum, mRNA expression of *VR1a* and *AQP11* was lower, and *REN*, *ATP1B1* and CLDN2 higher in LWCR compared to HWCR broilers. In the cecum, the LWCR line exhibited higher expression of *VR2*, *ACE*, and *CLDN15* and lower expression of *AGT* and *AQP*5 compared to HWCR broilers. Female broilers exhibited lower expression of *AQP4* in the upper duodenum and lower expression of *AGT, REN, ACE, AT1*, and *AT2* in the ileum compared to males. In the cecum, females had elevated levels of *MTR*, *AGT*, *ACE*, *AT1*, *AT2*, *AQP1,2,3,4*, and 11, and *OCLN* and *CLDN*2 in the cecum compared to males.

**Conclusion:**

The results of this study provide insight into water homeostasis gene expression in intestines of male and female broilers divergently selected for LWCR and HWCR. We hope this information will contribute to greater understanding of water homeostasis in broilers and help sustain the broiler industry as water scarcity issues continue worldwide.

## 1 Introduction

Water scarcity is clearly having a negative impact on poultry production worldwide (El-Sabry et al., 2023). The last decade has been the hottest since global temperatures have been recorded (Earth.org, 2026). Droughts are increasing the number and size of regions of water scarcity and placing water availability in jeopardy for billions of people and food supplies worldwide. A report by Liu et al. (2022) indicated that 19 major grain crops grown globally are likely to experience increased water scarcity of 80 to 90% by 2050. It is anticipated that high temperatures will become even more extreme and that the duration of these extreme temperature events will be longer (Xu et al., 2018; Tollefson, 2022) and will only magnify water scarcity issues facing the world.

Broilers are one of the most efficient animals in converting feed into high quality animal protein due to intensive selection for growth performance. Although there have been tremendous strides made in improving growth performance (body weight gain [BWG] and feed conversion ratio [g feed/g BWG, FCR]) in commercial broilers, little attention has been paid to improving water use efficiency (water conversion ratio [WCR, g water intake/g BWG]) due in part to the difficulty in measuring low water flow rates and the labor intense nature of accurately measuring water intake on individual birds. Broilers consume roughly 1.6 to 2.0 times more water than feed. A technical report indicated that from 1–35 days of age, broilers exhibit a water to feed ratio of approximately 1.8 (Aviagen, 2025). Thus, genetic selection for FCR has concomitantly reduced water intake per unit body weight. Residual water intake (RWI) has been used to measure water efficiency in broilers and cattle (Aggrey et al., 2023; Ahlberg et al., 2019) but to our knowledge genetic selection for water efficiency has not been conducted to date. Using a low water flow rate measuring device (Hiltz, 2021; Hiltz et al., 2021), broiler lines have been selected for high and low water efficiency, characterized as low water conversion ratio (LWCR) and high water conversion ratio (HWCR), respectively. The LWCR and HWCR lines were selected from a base control population of broilers, designated modern random bred (MRB) that was a composite of the commercial broiler genetics available in 2016 (Orlowski et al., 2020).

Water homeostasis (i.e. water balance) depends on water intake, the actions of several organs (e.g. hypothalamus, kidney, intestines), hormonal systems such as antidiuretic hormone and renin-angiotensin system, and membrane associated proteins such as aquaporins and tight junction proteins. Arginine Vasotocin (*AVT*) is the avian antidiuretic hormone that is released from the hypothalamus and stimulates water reabsorption in the collecting ducts of nephrons. Mesotocin (*MT*) is structurally similar to *AVT* and acts as a diuretic hormone, possibly by diverting blood flow in the avian kidney from mammalian type nephrons in the renal medulla to reptilian type nephrons in the cortical region (Robinzon et al., 1988). Actions of *AVT* and *MT* are mediated by binding to specific receptors; *VR1a* and *VR1b* (Selvam et al., 2015; Cornett et al., 2003), *VR2* (Tan et al., 2000), and mesotocin receptor (*MTR*) (Gubrij et al., 2005). Lower hypothalamic *AVT* mRNA expression in the LWCR line was hypothesized to play a role in reduced water intake in these broilers compared to the water inefficient (HWCR) line (Aloui et al., 2024). However, *AVT* mRNA expression in the kidney was elevated in LWCR broilers compared to the MRB line at both thermoneutral and heat-stress temperatures (Lassiter et al., 2025). Similarly, *AVT* mRNA expression was elevated in the ileum of the LWCR compared to MRB expression at thermoneutral temperatures (Lassiter et al., 2026). The elevation of *AVT* expression in the intestines during heat stress suggests that increased intestinal *AVT* expression could be involved in water homeostasis (Lassiter et al., 2026). Aquaporin proteins facilitate water movement between extracellular and intracellular compartments and between cells (e.g. Ishibashi et al., 2009). *AQP*1, 3 and 5 were upregulated in the LWCR line and heat stress elevated *AQP* 1 and 3 in the upper duodenum as well as *AQP*3 and 11 in the ileum (Lassiter et al., 2026). Based on fluorescein isothiocyanate-dextran (FITC) clearance from the blood, Greene et al. (2025) reported that water efficient LWCR broilers would lose less water into the ileum than water inefficient (HWCR) broilers under both thermoneutral and heat stress conditions. Greene et al. (2025) concluded that differential expression of key proteins and genes associated with gut epithelial cells would contribute to greater water use efficiency in the LWCR line as well as in the response to heat stress.

Using the unique broiler lines (LWCR, HWCR, and MRB), we have undertaken a series of studies to investigate growth performance and effects of heat stress on molecular expression associated with water homeostasis that include; 1) the avian antidiuretic hormone (arginine vasotocin, *AVT*) system, 2) the renin angiotensin (RAS) system, 3) members of the aquaporin (*AQP*) family, and 4) genes that encode tight junction proteins in the hypothalamus, kidney and intestines (Aloui et al., 2024; Greene et al., 2025; Lassiter et al., 2025, 2026). The study by Lassiter et al. (2026) investigated intestinal gene expression in male broiler progeny from the LWCR line compared to the MRB line on tissues obtained from randomly selected broilers in floor pens; i.e. birds were not individually phenotyped. Thus, the purpose of the present study was to determine mRNA expression in the intestines in both male and female broilers individually phenotyped for LWCR and HWCR that we have not previously reported.

## 2 Materials and methods

### 2.1 Ethics statement

The present study was conducted in accordance with the recommendations in the guide for the care and use of laboratory animals of the National Institutes of Health and the protocol was approved by the University of Arkansas Animal Care and Use Committee (protocol # 23015). Intestinal tissue samples in this study were obtained from a previous study (Lassiter et al., 2025) to reduce the number of animals used in our investigations into water efficiency; which is a desire expressed by federal funding agencies (e.g. USDA, NIH).

### 2.2 Phenotyping for LWCR and HWCR in male and female broilers

Procedures for phenotyping of broilers in this study were described previously (Hiltz et al., 2021; Lassiter et al., 2025). Briefly, eggs from LWCR and HWCR lines after the fourth generation of selection, were collected, incubated, and hatched at the University of Arkansas poultry hatchery. Each line is produced from 24 dams and 24 sires per line. On day of hatch, all chicks in this study were neck tagged, weighed, and randomly placed in floor pens (n=50) with clean pine shavings. During the brooding period, the chicks received standard age-appropriate corn-soy diets provided by a local breeding company *ad libitum.* Chicks were kept at thermoneutral temperatures of 33 °C at hatch that was reduced to 25 °C at 4 wk of age. The photoperiod was 24h light to 0h dark (24: 0) for wk 1, 23:1 for wk 2, and 18:6 for the rest of the study. The relative humidity was monitored and ranged from 30-40%. At 4 wk, 9 male and 9 female broilers per line were randomly selected from the floor pens and placed in cages (0.46 m x 0. 31m x 0.41 m). Each cage had nipple drinkers for individual monitoring of water intake and feeders for measuring feed intake. Body weights (BW, g) and feed intake (FI, g) for the two-week period were determined.

Growth performance (body weight [BW, g], body weight gain (BWG, g), feed intake [FI, g], feed conversion ratio (FCR), and water parameters (water intake [WI], water conversion ratio [WCR]) for LWCR and HWCR broilers in this study were obtained in a previous study (Lassiter et al., 2025). Water intake was measured using the water metering device described in Hiltz et al. (2021). FCR was calculated as g FI/g BW gain and water conversion ratio (WCR) was calculated as g water intake/g BW gain. After the phenotyping period (4 to 6 wk), broilers from each line and gender with the lowest and highest WCR (n=5/per line and gender out of the 9 that were phenotyped) were humanely euthanized by cervical dislocation. Tissue samples (0.5 – 1.0 g) were rapidly excised from the upper duodenum (adjacent to the pancreatic loop), the ileum (immediately distal to Meckel’s diverticulum), and the cecum, flushed with cold saline, flash frozen in liquid nitrogen, and kept at -80 C.

### 2.3 Total RNA extraction, reverse transcription, and quantitative real-time PCR

Total RNA extraction from the intestinal segments was conducted as previously described (Aloui et al., 2024; Greene et al., 2024) with modifications. Tissues were homogenized in 1.5 mL lysis buffer [10 mM Tris-HCl (pH 8.0), 140 mM NaCl, 1.5 mM MgCl2, 0.5% Igepal, 2 mM vanadyl ribonuleoside complex (VRC, ThermoFisher Scientific, Waltham, MA)] using Next Advance bullet blender gold bead homogenizer (Next Advance, Troy, NY). One-tenth of the lysate was added to 1 mL Trizol reagent (ThermoFisher Scientific, Waltham, MA) for total RNA isolation according to manufacturer’s recommendations. Total RNAs were treated with RQ1 DNase. RNA integrity and quality were evaluated using 1% agarose gel electrophoresis and RNA concentrations and purity were measured for each sample by Take 3 micro volume plate using Synergy HT multi-mode microplate reader (BioTek, Winooski, VT). For cDNA synthesis, total RNA (1 µg) was reverse transcribed using qScript cDNA SuperMix (Quanta Biosciences, Gaithersburg, MD) (20 µL total volume). The reverse transcription reaction was conducted at 42 °C for 30 min followed by a 5 min incubation at 85 °C. Real-time quantitative PCR (Applied Biosystems 7500 Real-Time PCR system) was performed using 5 µL of 10X diluted cDNA, 0.5 µM of each forward and reverse specific primer, and PowerUp SYBR Green Master Mix (ThermoFisher Scientific, Rockford, IL) in a total 20 µL as previously described (Dridi et al., 2012). Oligonucleotide primers specific for chicken aquaporins (*AQP*1, 2, 3, 4, 5, and 11), arginine vasotocin (*AVT*) and its related receptors (*VR1a, VR1b, VR2*), mesotocin (*MT*) and it’s receptor (*MTR*), renin-angiotensin system [renin, *REN*, angiotensinogen *AGT*, angiotensin I converting enzyme *ACE*, angiotensin II receptor type 1 and 2 *AT1* and *AT2*], and ribosomal 18S as a housekeeping gene are compiled in **Table 1**. The cycling parameters for the qPCR amplification were as follows: an initial incubation at 50 °C for 2 min, an initial denaturation step (95 °C, 10 min) followed by 40 cycles of denaturation (95 °C, 15 s) and annealing (58 °C, 1 min). Melting curve analysis was applied at the end of the amplification by using the dissociation protocol (Sequence Detection system) to exclude contamination with unspecific PCR products. The PCR products were also confirmed by 2% agarose gel which exhibit only one definite band of the predicted size and by base sequencing. There were no gel-detected bands for the negative controls where the RT products were omitted. Relative expressions of target genes were determined by the 2^−ΔΔCt^ method (Schmittgen and Livak, 2008).

**Table 1:** Oligonucleotide primers for quantitative reverse transcription polymerase chain reaction (qRT-PCR).

Gene	Accession number^a^	Primer sequence (5’ ➔ 3’)	Orientation	Product size, bp
*ACE*	NM_001167732.2	ACCCAACGAAGAAAAGAGCTATTTAT	Forward	69
		GCCGGTGCCTGAATTTCTC	Reverse	
*AGT*	NM_001396391.2	CAGGGTTTGCTGGGATTTGT	Forward	55
		CCCTGGAGGTGCAATTGG	Reverse	
*AQP1*	NM_001039453.2	GGGACATCTCCTTGCAATTGATTA	Forward	118
		GATTGGGCCAACCCAGAAGA	Reverse	
*AQP2*	NM_001292072	TTTGCAGCCTCCATGATGTG	Forward	56
		AGGACAGCCCGGGTGAA	Reverse	
*AQP3*	XM_424500	TGCTCCTGGTCCCTGACACT	Forward	58
		CTTTTGCCTTCCCATTGCA	Reverse	
*AQP4*	NM_001317827	CGCTCGCAGCAGC*AGT*AA	Forward	59
		ATGCTACCATGATGCTCTCACACT	Reverse	
*AQP5*	XM_001231780.7	TGGCCATCAATTCGCTCAAC	Forward	120
		TGCCATTCCTCCTGTTGTCC	Reverse	
*AQP11*	XM_015280878.4	ATGCTGGTGTTTGCAGGTGG	Forward	134
		CCTAAGCATGGTGCTATCC*AGT*	Reverse	
*AT1*	NM_205157.4	GCCTTAGCATCGACCGCTAT	Forward	62
		GGTACGTCGGATTCGTGACTT	Reverse	
*AT2*	XM_040670971.2	GGAAACCCTCCAGATCCTCTATACA	Forward	62
		GCGGCGAGCGTAACACA	Reverse	
*ATP1B1*	NM_205520	CGCGTGGAATGCAAGGA	Forward	62
		CCCTGGAAGCGGTCTTTGT	Reverse	
*AVP*	NM_205185	TCCGGGCACACTCAGCAT	Forward	81
		ATGTAGCAGGCGGAGGACAA	Reverse	
*AVPR1A*	NM_001110438.2	CCCCTGGGTCGACTCTGAA	Forward	126
		CTCTGCACGC*AGT*CCTGTAG	Reverse	
*AVPR1B*	NM_001031498.2	TTTTGTCATCGTGGTGGCCT	Forward	103
		GTCGGCGGACTCGTCATC	Reverse	
*AVPR2*	NM_001031479.2	TGGTCACCATTCTTCATCGCA	Forward	76
		TGGTGAATGCTGATCCTTC*AGT*	Reverse	
*CLDN2*	NM_001277622.1	CCCAGCTGATGGCAAAGG	Forward	61
		AGGCTGATGGCACCAAAAT*AGT*	Reverse	
*CLDN15*	XM_046898719.1	ATATACTCGAGGGCCCATGTT	Forward	77
		ACAGCAGCTTTGAATCCCAGA	Reverse	
		GGTGGCATCTCCTCGGTAAC	Reverse	
*MT*	XM_025150346.3	AGGAGAACTTCCTGCCAACG	Forward	110
		AAGCACACAGCCCTCACTG	Reverse	
*MTR*	NM_001031569.2	CCAAGATCCGGACGGTCAAA	Forward	130
		ATGAAGGGGGAGGCTTCCTG	Reverse	
*OCLN*	NM_205128.1	CGCAGATGTCCAGCGGTTA	Forward	59
		GTAGGCCTGGCTGCACATG	Reverse	
*REN*	XM_025143918.3	TGCCGGGTCTTTCCATCA	Forward	65
		GGCATTTTCCACCACTAG	Reverse	
18S	AF173612	TCCCCTCCCGTTACTTGGAT	Forward	60
		GCGCTCGTCGGCATGTA	Reverse	

Gene Abbreviations: *ACE*, angiotensin I converting enzyme; *AGT*, angiotensinogen; *AQP*, aquaporin; *AT1* &2, angiotensin receptor 1, 2; *ATP1B1*, sodium-potassium ATPase, subunit B1; *AVT*, arginine vasotocin; *VR1a, VR1b*, AVT receptor 1a, 1b; *CLND*2, 15, claudin 2, 15; *MT*, mesotocin; *MT*R, *MT* receptor; *OCLN*, occluding; *REN*, renin.

### 2.4 Statistics

Data were analyzed by two-way ANOVA with line (LWCR *vs.* HWCR) and sex (male *vs.* female) and their interaction as classification variables. If the ANOVA revealed significant effects, the means were compared by Tukey’s HSD multiple comparison test using the Graph Pad Prism version 9.00 for Windows (Graph Pad Software, La Jolla California, USA). If the interaction effect was not significant, the factor line and environment were analyzed separately using a student T test. Differences were considered significant at P < 0.05.

## 3 Results

### 3.1 Growth performance and WCR

Growth performance (body weight gain [BWG], feed intake [FI], feed conversion ratio [FCR] water intake [WI], and water conversion ratio [WCR]) data in individually phenotyped LWCR and HWCR male and female broilers are presented in **Figure 1**. These data were reported by Lassiter et al. (2025, **Table 2**) and are presented here using LWCR and HWCR designations for high water efficiency and low water efficiency broilers, respectively. Although there were no significant gender x line interaction effects for these parameters, there was a significant effect (P < 0.05) of gender for BWG, FI (P = 0.063) (**Figure 1A**), and WI (P = 0.045) (**Figure 1B**). There was higher (P< 0.05) for WI, WI: FI, and WCR in the HWCR compared to LWCR broilers.

**Figure 1:**
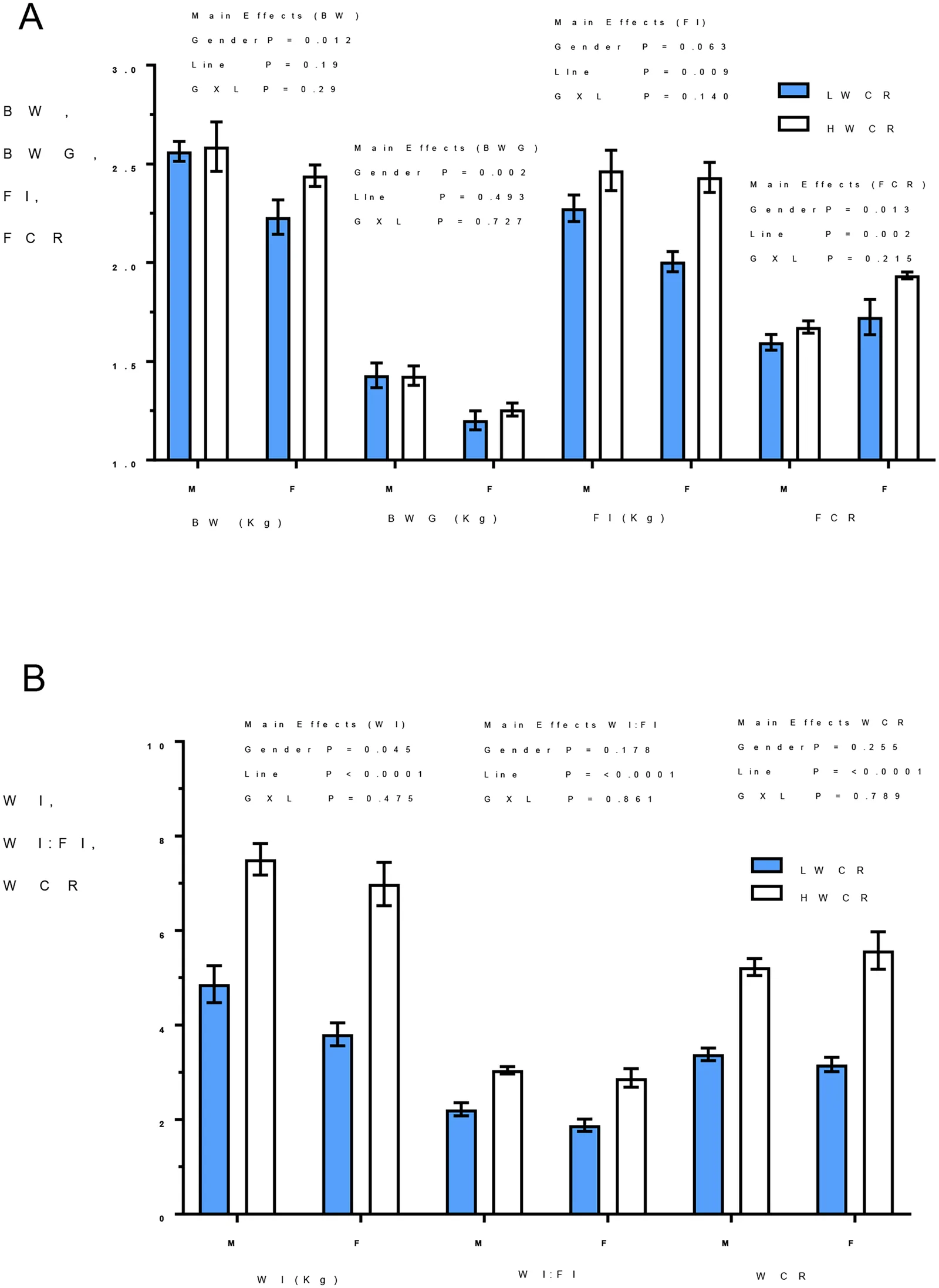
**(A)** Growth performance and **(B)** water coversion ratio (WCR) of individually phenotyped male (M) and female (F) broilers in the low (LWCR) and high (HWCR) broilers. Data (reported in Lassiter et al., 2025) are presented for body weight (BW, kg), 4–6 wk body weight gain (BWG, kg) feed intake (FI, kg), and feed conversion ratio (FCR) **(A)**; and water intake (WI, Kg), water intake: feed intake (WI: FI), and water conversion ratio (WCR) **(B)**. Main effect P values are presented for gender (G), line (L), and G x L interaction. Bars represent the mean ± SE (n=5).

**Table 2:** mRNA expression in upper duodenum for individually phenotyped male and female broilers from low water conversion ratio (LWCR) and high water conversion ratio (HWCR) lines (See **Table 1** for gene abbreviations).

Gene^1^	LWCR-Male				HWCR-Male				LWCR-Female				HWCR-Female				Line	Gender	L x G
	Mean	SE	n		Mean	SE	n		Mean	SE	n		Mean	SE	n		(L)	(G)	
*AVT*	1.00	0.16	5	ab	1.50	0.30	5	a	0.69	0.10	5	b	1.27	0.09	5	a	0.009	0.159	0.830
*VR2*	1.00	0.28	5	a	0.91	0.28	5	a	1.00	0.16	5	a	0.62	0.23	4	a	0.761	0.680	0.761
*VR1a*	1.00	0.26	5	ab	1.61	0.28	5	a	0.70	0.05	5	b	1.23	0.15	5	a	0.031	0.177	0.870
*VR1b*	1.00	0.25	4	a	0.66	0.16	3	a	1.41	0.24	3	a	1.26	0.36	4	a	0.397	0.095	0.739
*MT*	1.00	0.28	5	a	1.64	0.35	5	a	1.72	0.46	5	a	2.32	0.54	5	a	0.159	0.115	0.963
*MTR*	1.00	0.22	4	a	0.80	0.17	5	a	1.30	0.12	5	a	1.04	0.38	5	a	0.469	0.963	0.676
*AVT/MT*	1.00	0.53	5	a	0.56	0.15	5	a	0.25	0.05	5	a	0.35	0.07	5	a	0.347	0.104	0.347
*AGT*	1.00	0.27	5	ab	1.39	0.31	5	a	0.71	0.15	5	b	1.40	0.34	5	a	0.069	0.620	0.596
*REN*	1.00	0.07	5	a	1.10	0.23	5	a	0.86	0.23	5	a	0.94	0.31	5	a	0.670	0.519	0.966
*ACE*	1.00	0.08	5	a	1.20	0.20	5	a	0.46	0.15	5	b	1.29	0.20	4	a	0.007	0.192	0.076
*AT1*	1.00	0.28	5	a	0.96	0.20	5	a	0.62	0.11	5	a	0.88	0.26	5	a	0.628	0.317	0.510
*AT2*	1.00	0.21	5	a	0.99	0.18	5	a	0.63	0.11	5	a	0.92	0.22	5	a	0.460	0.252	0.430
*ATP1B1*	1.00	0.34	5	a	0.92	0.30	5	a	0.96	0.31	5	a	0.82	0.24	5	a	0.718	0.818	0.922
*AQP1*	1.00	0.21	5	ab	1.58	0.16	5	a	0.88	0.12	5	b	1.78	0.33	5	a	0.004	0.858	0.477
*AQP2*	1.00	0.25	5	a	0.62	0.12	5	a	0.62	0.17	5	a	0.61	0.06	5	a	0.257	0.256	0.280
*AQP3*	1.00	0.38	4	ab	1.35	0.15	5	a	0.78	0.12	5	b	1.22	0.16	5	ab	0.082	0.434	0.682
*AQP4*	1.00	0.26	5	a	0.59	0.10	5	ab	0.57	0.10	5	b	0.52	0.05	5	b	0.034	0.023	0.089
*AQP5*	1.00	0.13	5	a	0.96	0.13	5	a	0.74	0.17	5	a	1.07	0.06	5	a	0.277	0.568	0.171
*AQP11*	1.00	0.19	5	ab	1.31	0.09	5	a	0.84	0.18	5	b	1.22	0.04	5	a	0.025	0.385	0.806
*OCLN*	1.00	0.24	5	a	0.85	0.23	5	a	0.79	0.19	5	a	0.87	0.16	5	a	0.391	0.153	0.130
*CLDN2*	1.00	0.09	4	a	0.66	0.10	5	b	0.73	0.14	5	ab	0.51	0.11	5	b	0.028	0.087	0.610
*CLDN15*	1.00	0.19	5	a	1.54	0.61	3	ab	0.35	0.20	3	b	0.86	0.31	4	ab	0.130	0.056	0.963

Values represent the mean ± SE of ‘n’ observations.

abcd Means with different letters are different (P < 0.05).

### 3.2 Gene expression

Gene expression for upper duodenum, ileum, and cecum, for male and females from LWCR and HWCR lines, as well as computed main effects and interactions of the 2x2 factorial, are presented in **Tables 2**–**4**, respectively. The main effects for line (**Figure 2**) and gender (**Figure 3**) are based on relative expression values presented in **Supplementary Tables 1A–C** for the upper duodenum, ileum, and cecum, respectively.

**Table 3:** mRNA expression in ileum for individually phenotyped male and female broilers from low water conversion ratio (LWCR) and high water conversion ratio (HWCR) lines (See **Table 1** for gene abbreviations).

Gene^1^	LWCR-Male				HWCR-Male				LWCR-Female				HWCR-Female				Line	Gender	L x G
	Mean	SE	n		Mean	SE	n		Mean	SE	n		Mean	SE	n		(L)	(G)	
*AVT*	1.00 ±	0.18	5	a	1.48	0.26	5	a	1.14	0.21	5	a	1.04	0.14	5	a	0.3376	0.4406	0.1575
*VR2*	1.00 ±	0.37	5	a	1.09	0.15	5	a	1.29	0.03	5	a	1.16	0.24	5	a	0.9323	0.4488	0.6414
*VR1a*	1.00 ±	0.18	4	b	2.31	0.22	5	a	2.06	0.39	5	ab	2.38	0.36	4	a	0.0196	0.0892	0.1319
*VR1b*	1.00 ±	0.28	4	a	1.35	0.20	5	a	1.60	0.35	5	a	0.98	0.08	5	a	0.6365	0.6869	0.1046
*MT*	1.00 ±	0.17	5	a	0.96	0.19	5	a	0.86	0.26	5	a	0.82	0.18	5	a	0.8498	0.5102	0.9999
*MTR*	1.00 ±	0.30	5	a	0.67	0.18	4	a	0.87	0.20	4	a	0.48	0.14	4	a	0.1421	0.4995	0.8984
*AVT/MT*	1.00 ±	0.22	5	a	1.48	0.90	5	a	1.47	0.90	5	a	0.97	0.33	5	a	0.9752	0.9876	0.4505
*AGT*	1.00	0.24	4	a	0.82	0.10	5	ab	0.46	0.03	5	b	0.62	0.19	5	ab	0.9484	0.0279	0.2810
*REN*	1.00	0.1	4	a	0.62	0.05	5	b	0.51	0.07	5	bc	0.30	0.01	4	c	0.0002	<.0001	0.0659
*ACE*	1.00	0.31	4	a	0.58	0.11	5	ab	0.32	0.16	5	ac	0.24	0.04	5	c	0.1522	0.0077	0.3213
*AT1*	1.00	0.25	4	a	0.96	0.13	5	a	0.68	0.10	5	a	0.65	0.14	5	a	0.8304	0.0697	0.9756
*AT2*	1.00	0.15	5	ab	1.13	0.06	5	a	0.70	0.15	5	b	0.69	0.15	5	b	0.6587	0.0135	0.6068
*ATP1B1*	1.00	0.26	4	ab	0.54	0.05	5	b	1.17	0.30	4	a	0.55	0.11	5	ab	0.0120	0.6383	0.6758
*AQP1*	1.00	0.34	5	a	1.40	0.23	4	a	1.18	0.08	3	a	1.80	0.28	3	a	0.1130	0.3485	0.7174
*AQP2*	1.00	0.19	5	a	1.12	0.23	5	a	0.99	0.19	5	a	0.99	0.17	5	a	0.7637	0.7259	0.7367
*AQP3*	1.00	0.25	5	a	1.17	0.07	5	a	1.43	0.31	5	a	1.32	0.15	5	a	0.8911	0.1974	0.5254
*AQP4*	1.00	0.42	5	a	0.92	0.08	5	a	1.35	0.32	5	a	0.77	0.07	5	a	0.2370	0.7196	0.3658
*AQP5*	1.00	0.31	5	a	1.05	0.14	5	a	1.31	0.40	5	a	1.40	0.24	5	a	0.8115	0.2648	0.9456
*AQP11*	1.00	0.13	4	b	2.08	0.33	5	a	1.33	0.35	5	ab	2.16	0.35	5	a	0.0064	0.5104	0.6868
*OCLN*	1.00	0.03	4	ab	1.18	0.12	5	a	1.04	0.28	5	ab	0.71	0.07	5	b	0.6595	0.2170	0.1472
*CLDN2*	1.00	0.14	5	a	0.95	0.10	5	a	0.90	0.12	5	a	0.36	0.04	5	b	0.0139	0.0052	0.0356
*CLDN15*	1.00	0.41	4	a	0.42	0.17	4	a	0.60	0.19	4	a	0.52	0.18	5	a	0.2110	0.5602	0.3372

Values represent the mean ± SE of ‘n’ observations.

abcd Means with different letters are different (P < 0.05).

**Table 4:** mRNA expression in the cecum for individually phenotyped male and female broilers from low water conversion ratio (LWCR) and high water conversion ratio (HWCR) lines. (See **Table 1** for gene abbreviations).

Gene^1^	LWCR-Male				HWCR-Male				LWCR-Female				HWCR-Female				Line	Gender	L x G
	Mean	SE	n		Mean	SE	n		Mean	SE	n		Mean	SE	n		(L)	(G)	
*AVT*	1.00	0.24	4	a	1.10	0.18	5	a	1.33	0.29	5	a	1.37	0.14	5	a	0.876	0.247	0.772
*VR2*	1.00	0.12	4	a	0.49	0.06	5	c	1.12	0.03	5	a	0.70	0.06	5	b	0.000	0.030	0.523
*VR1a*	1.00	0.20	4	a	0.95	0.17	5	a	0.79	0.16	5	a	0.75	0.16	3	a	0.807	0.277	0.978
*VR1b*	1.00	0.15	4	a	0.99	0.08	5	a	1.10	0.18	5	a	1.08	0.12	5	a	0.915	0.500	0.972
*MT*	1.00	0.20	5	a	0.56	0.21	5	a	0.97	0.12	5	a	0.70	0.20	5	a	0.075	0.771	0.654
*MTR*	1.00	0.19	5	b	1.15	0.33	5	b	3.90	0.38	5	a	2.21	0.66	5	a	0.070	0.000	0.034
*AVT/MT*	1.00	0.40	4	b	1.24	0.17	5	b	2.33	1.48	4	ab	2.62	0.53	4	a	0.101	0.735	0.975
*AGT*	1.00	0.11	4	b	1.02	0.05	5	b	1.25	0.21	4	b	1.84	0.14	5	a	0.039	0.0013	0.052
*REN*	1.00	0.21	5	a	0.89	0.16	5	a	1.14	0.18	5	a	0.96	0.07	5	a	0.388	0.5300	0.833
*ACE*	1.00	0.19	4	b	0.72	0.03	4	bc	1.90	0.21	5	a	0.99	0.06	5	b	0.001	0.0016	0.054
*AT1*	1.00	0.03	4	b	0.94	0.09	5	b	1.56	0.31	5	ab	1.92	0.25	5	a	0.473	0.0020	0.320
*AT2*	1.00	0.09	4	bc	1.13	0.12	5	b	1.79	0.33	4	ab	2.17	0.15	5	a	0.191	0.0002	0.512
*ATP1B1*	1.00	0.22	4	a	0.80	0.21	5	a	1.03	0.25	5	a	0.79	0.06	5	a	0.987	0.9994	0.999
*AQP1*	1.00	0.27	4	b	1.11	0.15	5	b	2.38	0.24	5	a	2.69	0.27	5	a	0.388	<0.0001	0.678
*AQP2*	1.00	0.14	4	a	1.06	0.17	5	a	1.89	0.38	5	a	1.44	0.14	5	a	0.431	0.0187	0.306
*AQP3*	1.00	0.21	4	b	1.01	0.10	5	b	1.81	0.20	4	a	1.81	0.19	4	a	0.976	0.0002	0.976
*AQP4*	1.00	0.14	4	ab	0.85	0.09	5	b	1.22	0.16	5	ab	1.19	0.07	5	a	0.482	0.0415	0.482
*AQP5*	1.00	0.24	4	b	2.77	0.30	5	a	0.98	0.23	5	b	2.49	0.43	4	a	0.000	0.6464	0.691
*AQP11*	1.00	0.11	4	b	1.31	0.15	5	b	1.46	0.38	4	ab	1.74	0.11	5	a	0.169	0.0460	0.942
*OCLN*	1.00	0.1	4	ab	0.74	0.13	5	b	1.37	0.10	5	a	1.23	0.17	5	ab	0.063	0.0007	0.554
*CLDN2*	1.00	0.27	4	ab	1.40	0.25	5	ab	1.69	0.07	4	a	1.27	0.04	4	b	0.961	0.1862	0.062
*CLDN15*	1.00	0.35	4	ab	0.45	0.23	4	b	1.48	0.36	4	a	0.73	0.02	4	ab	0.366	0.1942	0.724

Values represent the mean ± SE of ‘n’ observations.

abcd Means with different letters are different (P < 0.05).

**Figure 2:**
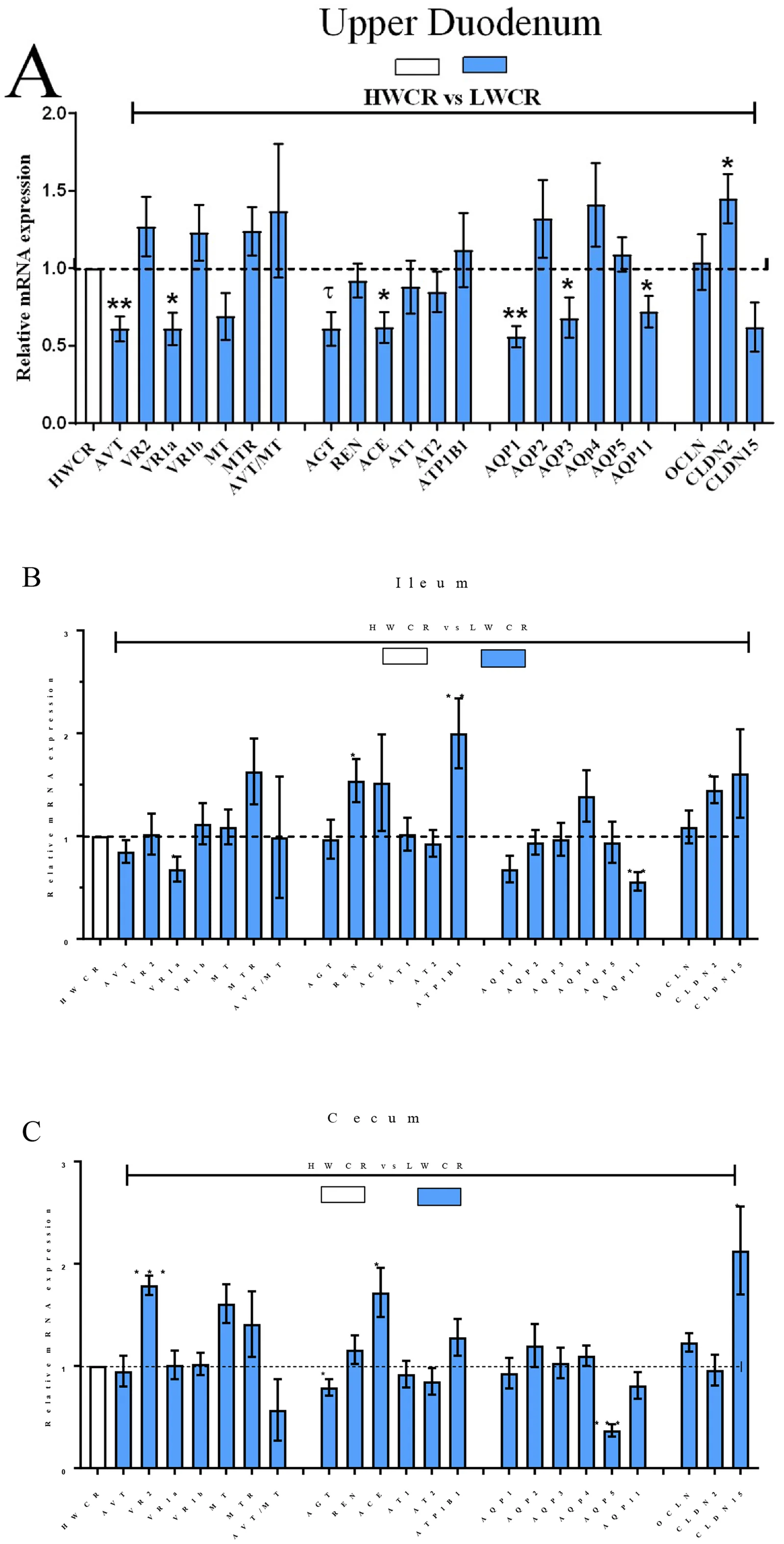
The main effect of broiler line (LWCR compared to HWCR) on relative gene expression for the AVTfamily (*AVT, VR2, VR1a, VR1b, MT, MTR and* AVT/MT), the RAS system (*AGT*, REN, *ACE*, *AT1*, *AT2*, and ATP1B1), *AQP* family (*AQP*1, 2, 3, 4, 5, and 11), and tight junction proteins (*OCLN*, *CLD2*, *CLDN*15) in the upper duodenum **(A)**, ileum **(B)** and cecum **(C)**. (Data for main effects are shown in **Supplementary Tables 1A–C**). Bars represent mean ± SE (n=7-10). Significance: *P < 0.05, **P < 0.01,***P < 0.001, τ P< 0.06.

**Figure 3:**
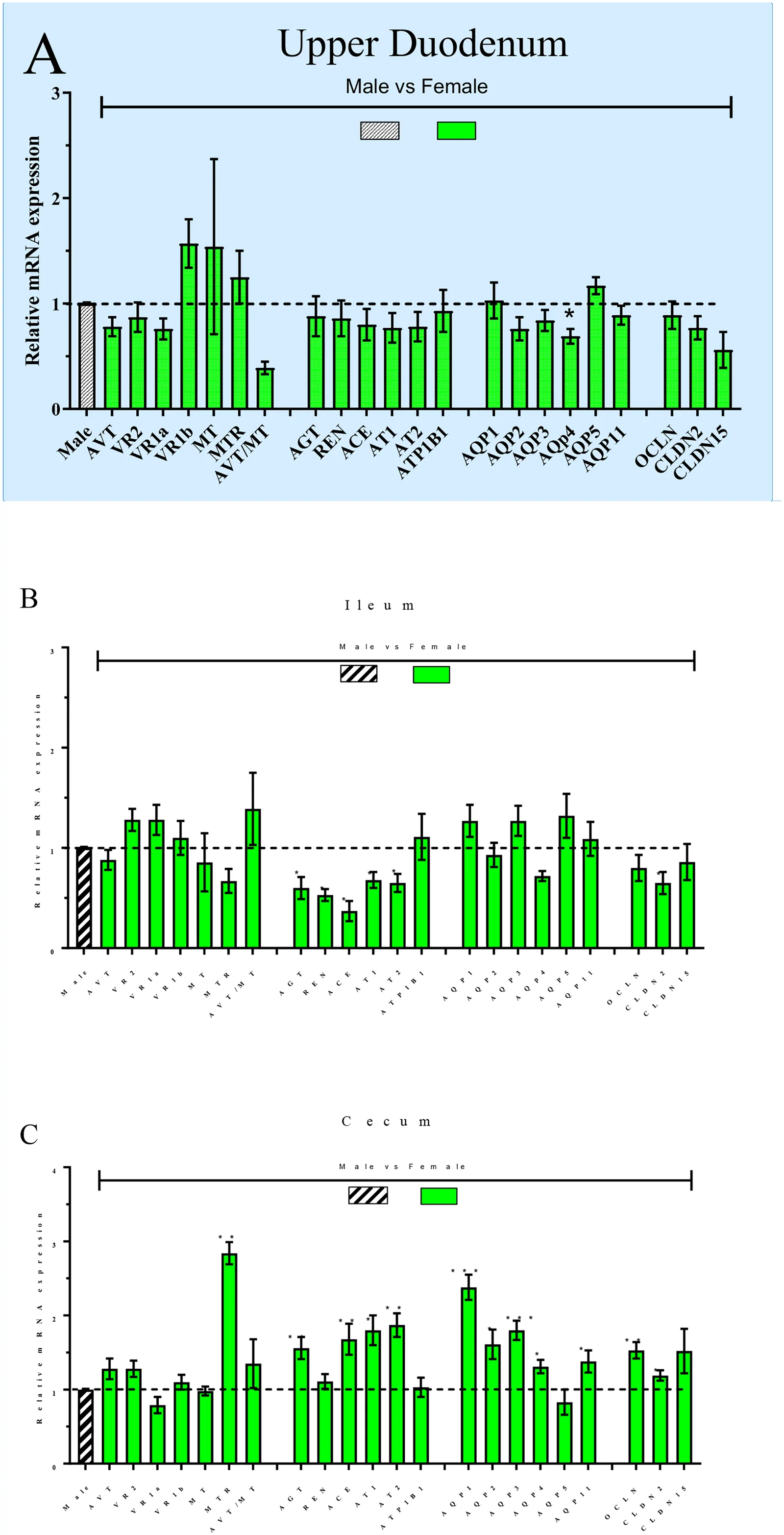
The main effect of gender (female vs male) on relative gene expression for the*AVT* family (*AVT, VR2, VR1a, VR1b*, *MT*, *MT*R and *AVT/MT*), the RAS system (*AGT*, *REN*, *ACE*, *AT1*, *AT2*, and *ATP1B1*), *AQP* family (*AQP*1, 2, 3, 4, 5, and 11), and tight junction proteins (*OCLN*, CLD2, *CLDN*15) in the upper duodenum **(A)**, ileum **(B)** and cecum **(C)**. (Data for main effects are shown in **Supplementary Tables 1A–C**). Bars represent mean ± SE (n=7-10). Significance: *P < 0.05, **P < 0.01, ***P < 0.001.

#### 3.2.1 Upper duodenum

In the upper duodenum, there were no differences in gene expression between LWCR and HWCR males with the exception that the expression of the tight junction encoding gene, *CLDN*2, was higher (P< 0.05) in LWCR males (**Table 2**). This contrasts with gene expression in female broilers in which *AVT, VR1a*, *AGT ACE*, *AQP*1 and *AQP*11 mRNA expression was higher (P< 0.05) in HWCR compared to LWCR females (**Table 2**). Statistical significance of main effects of line, gender, and line x gender interaction in the upper duodenum are also shown in **Table 2** with main effect mean values provided in **Figure 2**; **Supplementary Table 1A**. HWCR gene expression was higher (P ≤ 0.05) than in LWCR broilers in the upper duodenum for *AVT, VR1a*, *ACE*, *AQP1*, and *AQP*11, whereas *CLDN*2 was higher (P < 0.05) in LWCR broilers. There were no differences due to gender (with the exception of *AQP4* which was lower in females than in males). There was no line x gender interaction in gene expression in the upper duodenum (**Table 2**). The only main effect on protein expression due to gender in the upper duodenum was AQP 5 that was lower in females compared to males (**Figure 3A**).

#### 3.2.2 Ileum

There were no differences in gene expression in the ileum between LWCR males and HWCR males with the exception of higher expression of *VR1a and AQP11* that were higher in HWCR and *REN* that was higher in LWCR males (**Table 3**). There were also no differences in gene expression in female broilers between groups with the exception that *CLDN2* expression was higher in LWCR compared to HWCR females (**Table 3**). The only significant line x gender interaction in the ileum was observed for *CLDN*2 expression.

The main effect of broiler line on ileal gene expression is presented in **Figure 2B** based on values in **Supplementary Table 1B**. The results indicate that expression levels of *VR1a*, and *AQP11* were lower (P < 0.05), and that *REN* and *ATP1B1*, and *CLDN*2 were higher (P < 0.05) in the LWCR line compared to the HWCR line. Female broilers exhibited lower expression of *AGT*, *REN*, *ACE*, *AT1*, *AT2*, and *CLDN*2 in the ileum compared to males (**Figure 3B**; **Supplementary Table 1B**).

#### 3.2.3 Cecum

In the cecum, the only difference in gene expression in males between lines was lower expression of *VR2* and higher expression of *AQP*5 in HWCR males compared to LWCR males (**Table 4**). Differences between LWCR and HWCR females in the cecum included higher expression of *VR2* in LWCR females and higher expression of *AGT*, *ACE*, *AQP5*, and *CLDN*2 in HWCR females compared to LWCR females (**Table 4**). Main effects due to line included higher expression of *VR2*, *ACE*, and *CLDN15* and lower expression of *AQP*5 in the LWCR broilers compared to the HWCR line (**Figure 2C**; **Supplementary Table 1C**). Main effects due to gender included elevations in *MTR*, *AGT*, *ACE*, *AT1*, *AT2*, *AQP*1-4, *AQP*11, *OCLN*, and *CLDN*2 in females (**Figure 3C**; **Supplementary Table 1C**).

## 4 Discussion

### 4.1 Water use efficiency

A recent study in males and females from 4 commercial broiler strains provided WI, FI and WCR data from 0 to 56 days of age (Hiltz et al., 2025). Using this data, the average WI: FI ratio and WCR over 56 d across the strains and gender were 1.95 ± 0.02 and 3.31 ± 0.04, respectively. The WI: FI ratio from Hiltz et al. (2021) is very similar to that presented in a technical bulletin (Aviagen, 2025). In the present study mean WI: FI ratios study were 2.1 and 1.8 for LWCR males and females, respectively, compared to 3.0 and 2.9 in HWCR males and females. WI: FI ratios were 1.73 and 2.26 for water efficient (LWCR) male broilers raised in floor pens under thermoneutral or heat stress conditions from 4–7 wks compared to WI: FI ratios of 1.99 and 2.53 for water inefficient (HWCR) male broilers (Aloui et al., 2024). In the present study, LWCR broilers were more efficient than HWCR broilers for both feed and water utilization.

Aggrey et al. (2023) indicated that using residual water intake would differentiate low and high water efficiency broilers more accurately since WCR has the potential to be confounded by both WI and BWG. However, improved WCR in the LWCR group was independent of BW and BWG and was associated with better FCR. Thus, WCR does not appear to be confounded with BWG between LWCR to HWCR broilers in the present study. We will discuss gene expression results related to 4 different systems; 1) The *AVT* system (i.e. *AVT*, *MT* and their receptors), 2) the RAS system, 3) the *AQP* family, and 4) tight junction encoding as was reported previously in heat-stressed male broilers from the LWCR and the MRB lines (Lassiter et al., 2026).

### 4.2 AVT, MT, and receptors

In mammals, antidiuretic hormone (*AVP*) was hypothesized nearly 60 years ago to increase water secretion directly or by interfering with active Na+ transport (Soergel et al., 1968). Similar to mammals, the avian antidiuretic hormone (*AVT*) system in birds includes *AVT*, and its receptors, *VR2*, *VR1a* and *VR1b*. Birds also produce mesotocin (*MT*) that is structurally similar to *AVT* with only a single amino acid substitution (Robinzon et al., 1988). Both *AVT* and *MT* play roles in oviposition by binding to their receptors that are analogous to oxytocin receptors in mammals (Wu et al., 2019). Arroyo et al. (2022) reported that *AVP*, produced *de novo* in cortical collecting duct cells in the kidney, faciliated water reabsorption by a similar cascade mechanism as AVP arriving via systemic vasculature. We hypothesized that the presence of *AVT* and its receptors may also occur *de novo* in the intestines of broilers (Lassiter et al., 2026).

Aloui et al. (2024) reported that hypothlamic *AVT* mRNA expression was lower in water efficient broilers than in water inefficient broilers and hypothesized that this downregulation may contribute to lower water intake in the LWCR line. This finding differs from Aggrey et al. (2023) who observed higher expression of *AVT* in the hypothalamus of broilers that exhibited low residual water intake (i.e. water use efficient broilers). A difference in these studies may stem from the fact that broilers in Aloui et al. (2024) had been selected for water efficiency for 4 generations whereas broilers in the Aggrey et al. (2023) study were phenotyped for low residual water intake in a single group of broilers; i.e. these birds were not selected for WCR.

The results in the present study contrast with our previous one in which LWCR males exhibited up-regulation of *AVT* in the ileum, *VR2* upregulation in the upper duodenum and ileum, and up-regulation of *VR1a* and *VR1b* (upper duodenum only) compared to MRB broilers (Lassiter et al., 2026). *AVT* expression was also up-regulated by heat stress in all three intestinal segments (Lassiter et al., 2026). *AVT* and two of its main receptors, *VR2* and *VR1a*, were also up-regulated in the kidney in LWCR compared to HWCR male broilers (Lassiter et al., 2025) The finding of increased *AVT* in the kidney and the intestines in response to heat stress could be teleologically appropriate in helping animals cope with heat stress conditions. A difference between Lassiter et al. (2026) and the present study is that birds were raised in cages in the present study whereas broilers were raised in floor pens in Lassiter et al. (2026). Thus, we hypothesize that there might be an effect of litter in the previous study that resulted in differences in intestinal gene expression that merits further investigation.

### 4.3 Renin-angiotensin system (RAS)

The RAS system was recognized over 60 years ago to play an important role in gut physiology through its affects on gut motility (Robertson and Rubin, 1958, 1962). The RAS system is involved in intestinal physiology as indicated by the presence of genes, proteins, hormones, and hormone receptors throughout the gastrointestinal tract (Garg et al., 2012; Zizzo and Serio, 2023) and has been reported to interact with gut microbiota (Jaworska et al., 2021). However, according to Zizzo and Serio (2023) little research has been devoted to studying functionality of intestinal RAS in any species.

To our knowledge, there are few investigations of intestinal physiology and function of the RAS system carried out in avian species. As indicated by Garg et al. (2012), the classic action of the RAS system is to enhance renal reabsorption of Na^+^ and water that begins with stimulation of renin secretion in juxtamedullary cells of the kidney that enzymatically cleaves angiotensinogen (*AGT*) circulating in plasma (following its secretion from the liver) to form angiotensin I. Angiotensin I is then converted to Angiotensin II by angiotensin converting enzyme (*ACE*) found in endothelial cells. ANGII causes vasoconstriction of afferent arterioles in the kidney and stimulates aldosterone secretion from the adrenal gland that results in enhanced Na+ and water reabsorption in the kidney. ANGI can also be converted to Angiotensin (1-7) through activity of *ACE*2 and *ACE*. Whereas binding of ANGII to *AT1* receptors results in vasoconstriction and promotes inflammation and oxidation, binding of ANGII to *AT2* receptors can cause vasodilation, is anti-inflammatory, and has antioxidative activity (Garg et al., 2012).

There were few differences in RAS system gene expression between LWCR and HWCR lines in the present study. *AGT* and *ACE* were up-regulated in HWCR compared to LWCR females in the upper duodenum (**Table 2**). There were no differences in expression of any of the RAS components in the ileum between the 4 groups (**Table 3**). In the cecum, *AGT* was uprelated in HWCR females but *ACE* was down regulated in HWCR females compared to LWCR females (**Table 4**). With regard to main effects of line, compared to the HWCR line, a) the expression of *ACE* was lower in the upper duodenum in LWCR broilers (**Figure 2A**), b) the expression of REN and ATP1B1 were up-regulated in LWCR broilers (**Figure 2B**), and c) *AGT* was down-regulated and *ACE* being upregulated in the cecum (**Figure 2C**). There was a main effect of gender on several components of the RAS system with down-regulation of *AGT*, *REN*, *ACE*, *AT1*, and *AVT* in the females in the ileum (**Figure 3B**) but these were up-regulated (with the exception of REN) in the cecum in females compared to male broilers (**Figure 3C**). Additional studies on the RAS system including functional/physiological levels within the various layers of the gut (e.g. mucosa and serosa) will be required in order to determine specific roles RAS may have in the avian gut as well as in males and females in the LWCR and HWCR broiler phenotypes.

### 4.4 *AQP* family

Water can move across the gut epithelium either by transcellular routes through aquaporin channels or by paracellular routes that are regulated by tight junction proteins (Zhu et al., 2016). The 13 identified *AQP*s are placed in 3 group characteristics; those that are, 1) permeable to water alone (*AQP*0-*AQP*8), 2) permeable to water, glycerol, and urea (*AQP*3, *AQP*7-10), and 3) nonclassical (*AQP*11-12) (Ishibashi et al., 2009). Compared to the HWCR line, *AQP1*, *AQP*3 and *AQP11* were down-regulated in the upper duodenum in LWCR broilers (**Figure 2A**). These findings are opposite to elevated expression of *AQP* 1, 3, and 11 in the upper duodenum of LWCR compared to MRB broilers (Lassiter et al., 2026). Again, we think that the difference in the results might be due to differences in bird housing (cages in the present study vs floor pens in Lassiter et al. (2026). While there were no gender differences in expression in the duodenum or ileum, 5 of 6 members of the *AQP* family were up-regulated in females compared to males in the cecum (**Figure 3**). This would suggest that female broilers in this study might have an enhanced capability of water reabsorption from the cecum compared to males.

### 4.5 Tight junction proteins

Greene et al. (2025) reported that the LWCR line broilers have reduced water loss across the gut epithelium compared to HWCR broilers under thermoneutral and heat stress conditions. Genes of the claudin family that were up-regulated under thermoneutral conditions in the LWCR line that might contribute to decreased water movement into the lumen of the gut included *CLDN*1, *CLDN*4, and *CLDN*15. Other genes or proteins that would enhance tightly coupled epithelial cells in the ileum of the LWCR line included cingulin, gap junction A1, and desmosome G4 (Greene et al., 2025).

In the present study, we investigated 3 tight junction encoding genes; *OCLD*, *CLDN*2 and *CLDN*15. *CLDN*2 expression was up-regulated in the upper duodenum in LWCR compared to HWCR males (**Table 2**) and in the ileum and cecum of LWCR compared to HWCR females (**Tables 3**, **4**). A main effect of broiler line was evident with higher expression of *CLDN*2 in the upper and ileum (**Figures 2A, B**) and *CLDN*15 in the cecum (**Figure 2C**). We also observed increased expression of *CLDN*2 in the upper duodenum of LWCR male broilers in our previous study (Lassiter et al., 2026). These results support findings by Greene et al. (2025) who indicated a greater integrity of the epithelial tight junctions that would indicate greater tight junction integrity in the LWCR compared to the HWCR line. However, further studies will be required to further test this hypothesis.

### 4.6 Summary

In summary, this study is the first to report intestinal gene expression associated with water homeostasis in male and female broilers individually phenotyped for high and low water use efficiency (LWCR and HWCR, respectively). Water homeostasis mechanisms that were targeted included the *AVT* system, RAS (the renin angiotensin system), the *AQP* family, and three tight junction protein genes. Whereas a clear picture of differential expression of these groups of genes due to main effect of line (LWCR vs HWCR) was not observed (**Figure 2**), a main effect of gender was apparent with female broilers exhibiting a down regulation of the RAS system in the ileum, and an up-regulation of the RAS system and *AQP* family in the cecum of females compared to male broilers (**Figure 3**).

We currently have initiated transcriptomic, proteomic and metabolomic studies in several tissues that may be instrumental in delineating water homeostasis mechanisms more clearly in these unique broiler lines. However, additional studies utilizing a more mechanistic approachs will be needed in order to sort out all the physiological and molecular differences between immature male and female broilers in the LWCR and HWCR lines. For example, time course studies targeting specific proteins in the intestines as well as circulating hormones that could be combined with intestinal water and electrolyte flux and histological assessment of the intestinal tract, will be required to fully understand the differences in the RAS system and AQP family that has resulted from the divergent selection for water use efficiency. At the very least we hope this study and previous ones conducted on water use efficiency in LWCR and HWCR broilers will provide a roadmap for the development of genetic markers that might be used in commercial genetic programs to help improve water use efficiency in broilers worldwide.

## Data Availability

The original contributions presented in the study are included in the article/**Supplementary Material**. Further inquiries can be directed to the corresponding author.
