# Supplementary material for: Water homeostasis gene expression in intestines of male and female broilers divergently selected and individually phenotyped for low and high water conversion ratio
Source: Front Physiol. 2026 Oct 16;17:1954752. doi: 10.3389/fphys.2026.1954752 (PMC13719633; doi:10.3389/fphys.2026.1954752)
Supplement: Supplementary file 1 [file Table1.docx]

**Supplemental Table A. mRNA expression in Upper Duodenum. Main effects of sex (male and female) and line of broiler (LWCR and HWCR).**

| **Gene** | **Male** |  |  | **Female** |  |  |  | **HWCR** |  |  | **LWCR** |  |  |  |
| --- | --- | --- | --- | --- | --- | --- | --- | --- | --- | --- | --- | --- | --- | --- |
|  | **Mean** | **SE** | **n** | **Mean** | **SE** | **n** | **P Value** | **Mean** | **SE** | **n** | **Mean** | **SE** | **n** | **P Value** |
| **AVT** | 1.00 | 0.14 | 10 | 0.78 | 0.09 | 10 | 0.220 | 1.00 | 0.11 | 10 | 0.61 | 0.07 | 0.07 | 0.009 |
| **VPR2** | 1.00 | 0.20 | 10 | 0.87 | 0.14 | 9 | 0.604 | 1.00 | 0.22 | 9 | 1.27 | 0.19 | 0.19 | 0.360 |
| **VR1a** | 1.00 | 016 | 10 | 0.76 | 0.10 | 10 | 0.220 | 1.00 | 0.12 | 10 | 0.61 | 0.10 | 0.10 | 0.023 |
| **VR1b** | 1.00 | 0.19 | 9 | 1.57 | 0.23 | 9 | 0.088 | 1.00 | 0.23 | 7 | 1.23 | 0.19 | 0.19 | 0.446 |
| **MT** | 1.00 | 0.19 | 10 | 1.54 | 0.83 | 10 | 0.109 | 1.00 | 0.47 | 10 | 1.89 | 0.42 | 0.42 | 0.108 |
| **MTR** | 1.00 | 0.16 | 8 | 1.25 | 0.25 | 10 | 0.320 | 1.00 | 0.23 | 10 | 1.24 | 0.16 | 0.16 | 0.318 |
| **AVT/MT** | 1.00 | 0.36 | 10 | 0.39 | 0.06 | 10 | 0.090 | 1.00 | 0.59 | 10 | 1.37 | 0.63 | 0.63 | 0.567 |
|  |  |  |  |  |  |  |  |  |  |  |  |  |  |  |
| **AGT** | 1.00 | 0.18 | 10 | 0.88 | 0.19 | 10 | 0.642 | 1.00 | 0.15 | 10 | 0.61 | 0.11 | 0.11 | 0.057 |
| **REN** | 1.00 | 0.11 | 9 | 0.86 | 0.17 | 10 | 0.502 | 1.00 | 0.18 | 10 | 0.92 | 0.11 | 0.11 | 0.690 |
| **ACE** | 1.00 | 0.10 | 10 | 0.8 | 0.15 | 8 | 0.303 | 1.00 | 0.11 | 8 | 0.62 | 0.10 | 0.10 | 0.018 |
| **AT1** | 1.00 | 0.17 | 10 | 0.77 | 0.14 | 10 | 0.298 | 1.00 | 0.17 | 10 | 0.88 | 0.17 | 0.17 | 0.610 |
| **AT2** | 1.00 | 0.14 | 10 | 0.78 | 0.14 | 10 | 0.461 | 1.00 | 0.15 | 10 | 0.85 | 0.14 | 0.14 | 0.248 |
| **ATP1B1** | 1.00 | 0.22 | 10 | 0.93 | 0.20 | 10 | 0.810 | 1.00 | 0.20 | 10 | 1.12 | 0.25 | 0.25 | 0.700 |
|  |  |  |  |  |  |  |  |  |  |  |  |  |  |  |
| **AQP1** | 1.00 | 0.12 | 10 | 1.03 | 0.17 | 10 | 0.889 | 1.00 | 0.11 | 10 | 0.56 | 0.07 | 0.07 | 0.002 |
| **AQP2** | 1.00 | 0.18 | 10 | 0.76 | 0.11 | 10 | 0.261 | 1.00 | 0.11 | 10 | 1.32 | 0.25 | 0.25 | 0.260 |
| **AQP3** | 1.00 | 0.15 | 9 | 0.84 | 0.10 | 10 | 0.382 | 1.00 | 0.08 | 10 | 0.68 | 0.08 | 0.08 | 0.052 |
| **AQp4** | 1.00 | 0.19 | 10 | 0.69 | 0.07 | 10 | 0.129 | 1.00 | 0.10 | 10 | 1.41 | 0.27 | 0.27 | 0.170 |
| **AQP5** | 1.00 | 0.13 | 10 | 1.17 | 0.08 | 10 | 0.272 | 1.00 | 0.11 | 10 | 1.09 | 0.14 | 0.14 | 0.583 |
| **AQP11** | 1.00 | 0.10 | 10 | 0.89 | 0.09 | 10 | 0.437 | 1.00 | 0.04 | 10 | 0.72 | 0.10 | 0.10 | 0.021 |
|  |  |  |  |  |  |  |  |  |  |  |  |  |  |  |
| **OCLN** | 1.00 | 0.17 | 10 | 0.89 | 0.13 | 10 | 0.633 | 1.00 | 0.16 | 10 | 1.04 | 0.18 | 0.18 | 0.855 |
| **CLDN2** | 1.00 | 0.11 | 9 | 0.77 | 0.11 | 10 | 0.161 | 1.00 | 0.13 | 10 | 1.45 | 0.16 | 0.16 | 0.039 |
| **CLDN15** | 1.00 | 0.21 | 8 | 0.56 | 0.17 | 7 | 0.132 | 1.00 | 0.27 | 7 | 0.68 | 0.17 | 0.17 | 0.309 |

Values represent the mean ± SE of ‘n’ observations with P values shown for main sex and broiler line.
