# Supplementary material for: Water homeostasis gene expression in intestines of male and female broilers divergently selected and individually phenotyped for low and high water conversion ratio
Source: Front Physiol. 2026 Oct 16;17:1954752. doi: 10.3389/fphys.2026.1954752 (PMC13719633; doi:10.3389/fphys.2026.1954752)
Supplement: Supplementary file 2 [file Table2.docx]

**Supplemental Table B. mRNA expression in Lower Duodenum. Main effects of sex (male and female) and line of broiler (LWCR and HWCR).**

| **Gene** | **Male** |  |  | **Female** |  |  |  | **HWCR** | |  |  | **LWCR** |  |  |  |
| --- | --- | --- | --- | --- | --- | --- | --- | --- | --- | --- | --- | --- | --- | --- | --- |
|  | **Mean** | **SE** | **n** | **Mean** | **SE** | **n** | **P value** | | **Mean** | **SE** | **n** | **Mean** | **SE** | **n** | **P Value** |
| **AVT** | 1.00 | 0.14 | 10 | 0.88 | 0.10 | 10 | 0.493 | 1.00 | | 0.13 | 10 | 0.85 | 0.10 | 10 | 0.371 |
| **VR2** | 1.00 | 0.15 | 10 | 1.28 | 0.11 | 10 | 0.493 | 1.00 | | 0.12 | 10 | 1.02 | 0.20 | 10 | 0.929 |
| **VR1a** | 1.00 | 0.16 | 9 | 1.28 | 0.15 | 9 | 0.221 | 1.00 | | 0.08 | 8 | 0.68 | 0.12 | 9 | 0.044 |
| **VR1b** | 1.00 | 0.15 | 10 | 1.10 | 0.17 | 10 | 0.66 | 1.00 | | 0.10 | 10 | 1.12 | 0.20 | 9 | 0.602 |
| **MT** | 1.00 | 0.33 | 10 | 0.86 | 0.29 | 10 | 0.467 | 1.00 | | 0.15 | 10 | 1.09 | 0.23 | 10 | 0.831 |
| **MTR** | 1.00 | 0.33 | 9 | 0.67 | 0.12 | 8 | 0.265 | 1.00 | | 0.19 | 8 | 1.49 | 0.30 | 9 | 0.122 |
| **AVT/MT** | 1.00 | 0.70 | 10 | 1.39 | 0.36 | 10 | 0.31 | 1.00 | | 0.60 | 10 | 0.99 | 0.27 | 10 | 0.990 |
|  |  |  |  |  |  |  |  |  | |  |  |  |  |  |  |
| **AGT** | 1.00 | 0.13 | 9 | 0.6 | 0.11 | 10 | 0.026 | 1.00 | | 0.15 | 10 | 0.97 | 0.18 | 9 | 0.908 |
| **REN** | 1.00 | 0.10 | 9 | 0.53 | 0.06 | 9 | 0.002 | 1.00 | | 0.12 | 10 | 1.54 | 0.22 | 9 | 0.049 |
| **ACE** | 1.00 | 0.21 | 10 | 0.37 | 0.10 | 10 | 0.026 | 1.00 | | 0.20 | 10 | 1.52 | 0.47 | 10 | 0.320 |
| **AT1** | 1.00 | 0.13 | 9 | 0.68 | 0.08 | 10 | 0.048 | 1.00 | | 0.13 | 10 | 1.02 | 0.15 | 9 | 0.910 |
| **AT2** | 1.00 | 0.08 | 10 | 0.65 | 0.09 | 10 | 0.009 | 1.00 | | 0.11 | 10 | 0.93 | 0.13 | 10 | 0.691 |
| **ATP1B1** | 1.00 | 0.17 | 9 | 1.11 | 0.23 | 10 | 0.719 | 1.00 | | 0.10 | 10 | 2.00 | 0.34 | 9 | 0.007 |
|  |  |  |  |  |  |  |  |  | |  |  |  |  |  |  |
| **AQP1** | 1.00 | 0.18 | 8 | 1.27 | 0.16 | 6 | 0.320 | 1.00 | | 0.12 | 7 | 0.68 | 0.18 | 8 | 0.096 |
| **AQP2** | 1.00 | 0.13 | 10 | 0.93 | 0.12 | 10 | 0.706 | 1.00 | | 0.13 | 10 | 0.94 | 0.12 | 10 | 0.758 |
| **AQP3** | 1.00 | 0.12 | 10 | 1.27 | 0.15 | 10 | 0.179 | 1.00 | | 0.07 | 10 | 0.97 | 0.17 | 10 | 0.889 |
| **AQp4** | 1.00 | 0.18 | 10 | 0.72 | 0.05 | 10 | 0.669 | 1.00 | | 0.07 | 10 | 1.39 | 0.25 | 10 | 0.150 |
| **AQP5** | 1.00 | 0.16 | 10 | 1.32 | 0.22 | 10 | 0.248 | 1.00 | | 0.12 | 10 | 0.94 | 0.20 | 10 | 0.811 |
| **AQP11** | 1.00 | 0.16 | 9 | 1.09 | 0.17 | 10 | 0.704 | 1.00 | | 0.11 | 10 | 0.56 | 0.09 | 9 | 0.007 |
|  |  |  |  |  |  |  |  |  | |  |  |  |  |  |  |
| **OCLN** | 1.00 | 0.06 | 9 | 0.8 | 0.13 | 10 | 0.208 | 1.00 | | 0.11 | 10 | 1.09 | 0.16 | 9 | 0.667 |
| **CLDN2** | 1.00 | 0.09 | 10 | 0.65 | 0.11 | 10 | 0.02 | 1.00 | | 0.17 | 10 | 1.45 | 0.13 | 10 | 0.052 |
| **CLDN15** | 1.00 | 0.32 | 9 | 0.86 | 0.18 | 10 | 0.701 | 1.00 | | 0.24 | 10 | 1.61 | 0.44 | 8 | 0.224 |

Values represent the mean ± SE of ‘n’ observations with P values shown for main sex and broiler line.
