# Supplementary material for: Water homeostasis gene expression in intestines of male and female broilers divergently selected and individually phenotyped for low and high water conversion ratio
Source: Front Physiol. 2026 Oct 16;17:1954752. doi: 10.3389/fphys.2026.1954752 (PMC13719633; doi:10.3389/fphys.2026.1954752)
Supplement: Supplementary file 3 [file Table3.docx]

**Supplemental Table C. mRNA expression in Cecum. Main effects of sex (male and female) and line of broiler (LWCR and HWCR).**

| **Gene** | **Male** |  |  | **Female** |  |  |  | **HWCR** |  |  | **LWCR** |  |  |  |
| --- | --- | --- | --- | --- | --- | --- | --- | --- | --- | --- | --- | --- | --- | --- |
|  | **Mean** | **SE** | **n** | **Mean** | **SE** | **n** | **P Value** | **Mean** | **SE** | **n** | **Mean** | **SE** | **n** | **P Value** |
| **AVT** | 1.00 | 0.13 | 9 | 1.28 | 0.14 | 10 | 0.167 | 1.00 | 0.10 | 10 | 0.95 | 0.15 | 9 | 0.797 |
| **VR2** | 1.00 | 0.15 | 9 | 1.28 | 0.11 | 10 | 0.144 | 1.00 | 0.09 | 10 | 1.79 | 0.09 | 9 | 0.000 |
| **VR1a** | 1.00 | 0.13 | 9 | 0.79 | 0.11 | 8 | 0.249 | 1.00 | 0.12 | 8 | 1.01 | 0.14 | 9 | 0.970 |
| **VR1b** | 1.00 | 0.07 | 9 | 1.10 | 0.10 | 10 | 0.442 | 1.00 | 0.06 | 10 | 1.02 | 0.11 | 9 | 0.884 |
| MT | 1.00 | 0.21 | 10 | 0.98 | 0.06 | 10 | 0.940 | 1.00 | 0.24 | 10 | 1.61 | 0.21 | 10 | 0.058 |
| MTR | 1.00 | 0.176 | 10 | 2.84 | 0.149 | 10 | 0.001 | 1.00 | 0.24 | 10 | 1.41 | 0.32 | 10 | 0.320 |
| **AVT/MT** | 1.00 | 0.25 | 8 | 1.35 | .33 | 8 | 0.310 | 1.00 | 0.25 | 9 | 0.57 | 0.11 | 8 | 0.310 |
|  |  |  |  |  |  |  |  |  |  |  |  |  |  |  |
| **AGT** | 1.00 | 0.05 | 9 | 1.56 | 0.15 | 9 | 0.002 | 1.00 | 0.11 | 10 | 0.79 | 0.13 | 8 | 0.150 |
| **REN** | 1.00 | 0.13 | 10 | 1.11 | 0.10 | 10 | 0.525 | 1.00 | 0.09 | 10 | 1.16 | 0.14 | 10 | 0.360 |
| **ACE** | 1.00 | 0.12 | 9 | 1.68 | 0.21 | 10 | 0.018 | 1.00 | 0.07 | 9 | 1.72 | 0.24 | 9 | 0.010 |
| **AT1** | 1.00 | 0.05 | 9 | 1.80 | 0.20 | 10 | 0.002 | 1.00 | 0.15 | 10 | 0.92 | 0.13 | 9 | 0.684 |
| **AT2** | 1.00 | 0.07 | 9 | 1.87 | 0.16 | 10 | 0.0001 | 1.00 | 0.12 | 19 | 0.85 | 0.14 | 9 | 0.399 |
| **ATP1B1** | 1.00 | 0.16 | 9 | 1.03 | 0.13 | 10 | 0.887 | 1.00 | 0.13 | 10 | 1.28 | 0.18 | 9 | 0.212 |
|  |  |  |  |  |  |  |  |  |  |  |  |  |  |  |
| **AQP1** | 1.00 | 0.13 | 9 | 2.38 | 0.17 | 10 | 0.000 | 1.00 | 0.17 | 10 | 0.93 | 0.16 | 9 | 0.748 |
| **AQP2** | 1.00 | 0.10 | 9 | 1.61 | 0.20 | 10 | 0.020 | 1.00 | 0.10 | 10 | 1.20 | 0.22 | 9 | 0.384 |
| **AQP3** | 1.00 | 0.10 | 8 | 1.80 | 0.13 | 9 | 0.000 | 1.00 | 0.12 | 9 | 1.03 | 0.15 | 8 | 0.870 |
| **AQp4** | 1.00 | 0.08 | 9 | 1.31 | 0.09 | 10 | 0.021 | 1.00 | 0.08 | 10 | 1.10 | 0.11 | 9 | 0.437 |
| **AQP5** | 1.00 | 0.18 | 9 | 0.83 | 0.17 | 9 | 0.512 | 1.00 | 0.09 | 9 | 0.37 | 0.06 | 9 | 0.000 |
| **AQP11** | 1.00 | 0.09 | 9 | 1.38 | 0.15 | 9 | 0.044 | 1.00 | 0.12 | 10 | 0.81 | 0.13 | 8 | 0.200 |
|  |  |  |  |  |  |  |  |  |  |  |  |  |  |  |
| **OCLN** | 1.00 | 0.11 | 9 | 1.53 | 0.11 | 10 | 0.004 | 1.00 | 0.14 | 10 | 1.23 | 0.10 | 9 | 0.188 |
| **CLDN2** | 1.00 | 0.15 | 8 | 1.19 | 0.07 | 9 | 0.331 | 1.00 | 0.10 | 9 | 0.96 | 0.15 | 8 | 0.847 |
| **CLDN15** | 1.00 | 0.31 | 8 | 1.52 | 0.30 | 8 | 0.245 | 1.00 | 0.21 | 8 | 2.13 | 0.43 | 8 | 0.030 |

Values represent the mean ± SE of ‘n’ observations with P values shown for main sex and broiler line.
